# Effect of nest composition, experience and nest quality on nest-building behaviour in the Bonelli’s Eagle

**DOI:** 10.1038/s41598-022-08028-z

**Published:** 2022-03-09

**Authors:** José E. Martínez, Íñigo Zuberogoitia, José F. Calvo, Mario Álvarez, Antoni Margalida

**Affiliations:** 1 Departamento de Ecología E Hidrología, Universidad de Murcia, Murcia, Spain; 2 Bonelli’s Eagle Study and Conservation Group, Murcia, Spain; 3 Estudios Medioambientales Icarus, S.L. C/ San Vicente, 8. 6ª Planta. Dpto 8. Edificio Albia I, 48001 Bilbao, Spain; 4 Institute for Game and Wildlife Research, IREC (CSIC-UCLM-JCCM), Ciudad Real, Spain; 5 Division of Conservation Biology, Institute of Ecology and Evolution, University of Bern, Bern, Switzerland

**Keywords:** Ecology, Zoology

## Abstract

In bi-parentally built nests, there is evidence to suggest that nests are extended phenotypic signals that accurately indicate the quality of the building parent/s. Raptors often use a variety of materials to build their nests (natural, such as branches, but also non-natural objects), presumably due to their insulating properties, their suitability to advertise occupancy of the nest, and to decrease pathogen and parasite loads. However, in raptors where both sexes collaborate in nest construction, it is unclear whether nest building (taking the amount of material carried to the nest as the potential predictor) is an indicator of parental quality, and whether the effort expended by both sexes could constitute an honest signal of parental quality to their partners. Between 2011 and 2016, we monitored 16 nests of Bonelli’s Eagles (*Aquila fasciata*), and we examined data on sex, type of material brought to the nest, breeding experience, nest quality, timing, and nest-building investment prior to egg-laying from 32 identifiable Bonelli’s Eagles during the pre-laying period to investigate the relative contribution of the sexes to the amount of nest material gathered. Our results indicate that sex is not a determining factor in nest-building effort, and that females did not increase their parental effort in response to the male’s contribution, and supply of materials did not increase during the pre-laying period. In contrast, our models showed that: (1) the type of material supplied to the nest by both sexes varied significantly throughout the pre-laying period and (2) nest-building effort was determined by individual experience and nest quality. Therefore, our study suggests that male nest-building behaviour and investment by Bonelli’s Eagles cannot be considered as an extended phenotypic signal. The differential use of hard and green material by both sexes in the early and late stages of nest-building period, and the fact that the more experienced individuals contributed a larger amount of material on low quality nests, are discussed in the contexts of signaling nest occupancy to conspecifics and competitors and the decrease of ectoparasite loads during the pre-laying period.

## Introduction

Many animals use a wide variety of materials, designs, nest-site, and building techniques to build nests for shelter and reproduction^1–3^. Nests are essential structures for reproduction in some mammals and the majority of birds, and provide shelter and protection from inclement weather and predators for the eggs and nestlings^4,5^. Unlike animals that do not build their own nests, instead using the abandoned nests of heterospecifics to lay eggs and rear their young^6,7^, many species expend considerable time and energy in the construction of one or multiple elaborate nests for breeding^8,9^, with varying degrees of differential parental investment (i.e., exclusively maternal, paternal, or biparental; see^10^). These costs are even greater in eagles, since each pair builds or maintains multiple nests, alternating them in different years^11,12^.

Nest-building behaviour has been associated with courtship and pair formation because both sexes can use nest sites and nest material to attract a partner^13^. Nest-building behaviour also could provide information to an individual about the quality of a mate, and such assessment may also allow partners to invest differentially in reproduction relative to the quality of a mate^13^. Thus, nest-building activity could be used as sexually selected display^14–16^.

Avian nests are often concealed and camouflaged^2^, but many species build prominent nests or use conspicuous materials for nest ornamentation^17^. Previous studies have suggested that birds and mammals select materials for nest-building based on their thermal or structural properties^18^, which provide important benefits for the eggs and young^19,20^. For example, the use of feathers, fresh fragments of aromatic plants, or even cigarette butts as nest materials can play a key role reducing the adverse effects of pathogenic bacteria and parasites on eggshells^21^ and nestlings^22,23^, so improving the growth and condition of chicks at fledging^24^ to increase parental breeding success^12,25^. These direct benefits alone often make it beneficial to choose mates most able to build well-constructed nests^4,26,27^.

In some bird species, the nest size or the nest-building effort are considered as a reliable signal of parental ability^13,28,29^. In others, such as storks that reuse the same nest every breeding season, nest size increase steadily throughout the breeding period and only ceased when reproductive attempt failed or finished^9^. In this sense, in bi-parentally built nests, an increase in nest building effort over time would be expected^9^. In raptors, and mainly in eagles, the carrying of different material types to the nest begins during the courtship period, but the supply of material often continue throughout the incubation and chick-rearing periods^11^. However, in these species, little is known on the factors affecting the temporal variation in the amount and types of material selected by each sex in the construction of the nests. At the same time, the type of nest material can provide indirect benefits for birds. For example, during bird courtship displays, the degree of nest decoration and the speed and/or efficiency of gathering and transporting nest material may have additional or complementary functions, such as signaling: the extended phenotype of mate quality^30–32^; genetic quality^16^; nest-site occupancy; social status to potential intruders^17^; breeding experience^33,34^ and the willingness to attract partners and to invest in reproduction^13,35^. The nest-building experience of a mate may be an important factor influencing how and where nests are built in order to improve breeding success, because their previous experience enables them to optimize the nest characteristics to their particular requirements. Previous experience can influence decisions relating to the choice of nest-material in captive zebra finches^33,36^. In addition, experience in nest-building plays an important role in decision making in future building endeavors, because a bird will have had practice in how to use different materials from its environment to build a nest or to increase the speed of construction^37^. Therefore, in this line, older and more experienced mates could gather more material to the nest than youngers.

Previous research has shown that male displays of nest-building ability act as inter-sexual signals to attract females, mainly by the addition of green materials, flowers, feathers, and even stones to the nest^14,25,38^. In addition, female nest-building effort is determined by male physical attributes^13,16^. Both sexes contribute to nest-building in many different avian species^39^. Because nest-building is a costly activity in terms of both time and energy, and has fitness consequences, it would pay females to encourage male nest-building behaviour^5^. In this way, the number and size of items gathered to build nests could provide females with information on male condition or willingness to invest in reproduction^13^. To date, two hypotheses have been postulated to explain why females increase their parental effort when caring for the offspring of attractive males: (1) the partner-compensation hypothesis (PCH) postulates that females mated to attractive males elevate their own level of care to achieve increased reproductive success^40,41^ and (2) the differential allocation hypothesis (DAH) postulates that females mated to more attractive males are willing to contribute greater levels of parental investment compared with females mated to less attractive males^30,42^.

While investigations into extended phenotypic signals have been principally carried out on a variety of passerine species^43^, this topic has been scarcely explored in other avian groups, such as raptors^44^. Previous studies documenting the supply of nest-material to cliff nests by several raptor species have focused on: the functional aspects of decreasing ectoparasite loads in nests^12,45^; indicators of nest-site selection criteria^46^ social dominance; territory quality; means of signaling nest occupancy^17^; and indicators of the evolutionary load of past tree-nesting behaviour^47^. Although the parental behaviour of both sexes of Bonelli’s Eagle (*Aquila fasciata*) during breeding has been recently studied^48^, the role of their nest-building behaviour has been generally poorly explored. Bonelli’s Eagles build multiple large nests which they use alternately between years^12^. Bonelli’s Eagle nests consist of a large basal structure of hard materials (sticks) and a nest cup lined with the green branches of aromatic trees and shrubs, and other materials such as fresh and dead grasses^49^. Both sexes invest effort to repair one of the nests or build a new one throughout the 3–4 month period before egg-laying, although females may build while males gather nest-material^49^. This raises, on the first hand, the possibility that nest-building per se can be used as a signal of quality by both sexes, and they could benefit from mating with good nest-building partners. In this way, only individuals with high ability or good physical condition should be able to build large nests^13^. On the other hand, other factors that may determine nest-building behaviour and therefore parental investment are nest characteristics, for example, the nesting-support quality^50^. In nest reusers species, nest quality is related to the number of times that it was used in the past^9^, therefore, those nests used more frequently could be considered of high quality compared to the nests barely used, which in turn would be considered of low quality. These quality differences could strongly condition the final decision of the builders to select certain nests and may be related to the amount of material provided by individuals based on their potential ability. In this context, if nest building effort is related to bird experience and nest quality, one would expect that more experienced individuals (with presumably greater ability in nest building activity) would contribute more materials to the nests used more regularly (high quality nests), as nest size has been often used as a surrogate of nest quality^51^.

The objectives of our study were: (1) to assess whether the nest-building behaviour is an indicator of parental quality in Bonelli’s Eagles during the pre-laying period; (2) to assess a possible relationship between the parents’ nest-building effort and a set of explanatory variables (sex, week, nest material type, experience and nest quality); and (3) to investigate a possible relationship between male’s effort and productivity in this species. We hypothesize that (1) there are sex differences in nest building effort; (2) the amount of material contributed to the nest increases as the pre-laying period progresses; (3) the amount of the type of material contributed to the nest varies throughout the pre-laying period and there are differences in the types of materials supplied to the nests between sexes; (4) the nest-building effort is determined by the experience of individuals and the nest quality; and (5) males’ effort could act as an honest signal informing females about their parental quality; and females could increase their reproductive investment conditioned by the males’ behaviour.

## Material and methods

### Study species

Our model species, the Bonelli’s Eagle, is a large-sized eagle which inhabits mountainous areas across the Palearctic, Indo-Malayan and, marginally, the Afro-tropical regions^52^. It is a long-lived, sexually dimorphic species, and is monogamous and territorial. It builds its own nests using materials gathered by both sexes before egg-laying^53^. Bonelli’s Eagles build large, open, perennial nests mainly on cliffs and sometimes in trees^54^, with platforms composed of tree and shrub sticks and branches that can be reused for several years, or even decades, as new materials are added every breeding season^12,53^. The existence of alternative nests is often associated with competitive exclusion and ectoparasite deterrence^55^.

Bonelli’s Eagle is currently considered Endangered in Spain^56^ and as of Least Concern worldwide^57^. Their clutch size ranges from one to three eggs (less than 1% of clutches having three eggs^58^). Their diet is generalist, based principally on the European Wild Rabbit (*Oryctolagus cuniculus*) complemented with medium-sized birds such as pigeons (*Columba* spp.), Red-legged Partridges (*Alectoris rufa*) and corvids, as well as Ocellated Lizards (*Timon lepidus*)^53^. Regarding its reproductive biology, there is detailed information on parental investment of both sexes during breeding^48^. This last study revealed a marked division in parental duties in Bonelli’s Eagles: females invested more effort in incubation, nest attendance, chick-feeding and nest-building, while males contributed more to the provision of food to offspring.

### Study area

Our study was carried out in a large area in the Murcia and Almería provinces, southeastern Spain (37° 59′ N, 1° 29′ W). The climate is typically Mediterranean, with mean annual rainfall ranging from 200–400 mm. The vegetation consists of scrubland with small patches of Aleppo Pine (*Pinus halepensis*), interspersed with non-irrigated and irrigated crops in the foothills, plains, and valleys (for more details see^59^).

### Field work

Our study population consisted of 16 Bonelli’s Eagle pairs, nesting on cliffs at altitudes of up to 900 m. Between 2011 and 2016, 16 nests were monitored from October to one week after egg-laying (between January and February): six nests were observed in 2011, seven in 2012, two in 2014 and one in 2016. The nests were checked every seven days, monitoring them during overall daylight period. On each intensive monitoring day, we recorded the nest construction activity during the daylight hours from 06:00–18:00, the shortest observation period being 4 h (interrupted due to adverse weather) and the longest being 12 h. In total, 4131 h of nest monitoring were carried out using 20–60× telescopes, from points overlooking the territories at a distance of about 600–800 m from the nest. This distance did not appear to alarm the birds or affect their behaviour^59^.

Nest visits were recorded during each observation period, regardless of whether males or females arrived with (once branch or stick) or without nest-material^60^. At each nest visit, we recorded: (1) the type of material brought to the nest, whether fresh material (mainly green branches used for building the nest structure and decoration, and lining the interior of the nest) or hard material (mainly withered branches and sticks used for building the nest structure); (2) the date and time of material supply to the nest; (3) the number of branches or sticks supplied/individual/day (nest-building effort); (4) the return of a previously identified individual to a specific nest; and (5) the sex of an individual.

### Individual identification

**Figure 1:**
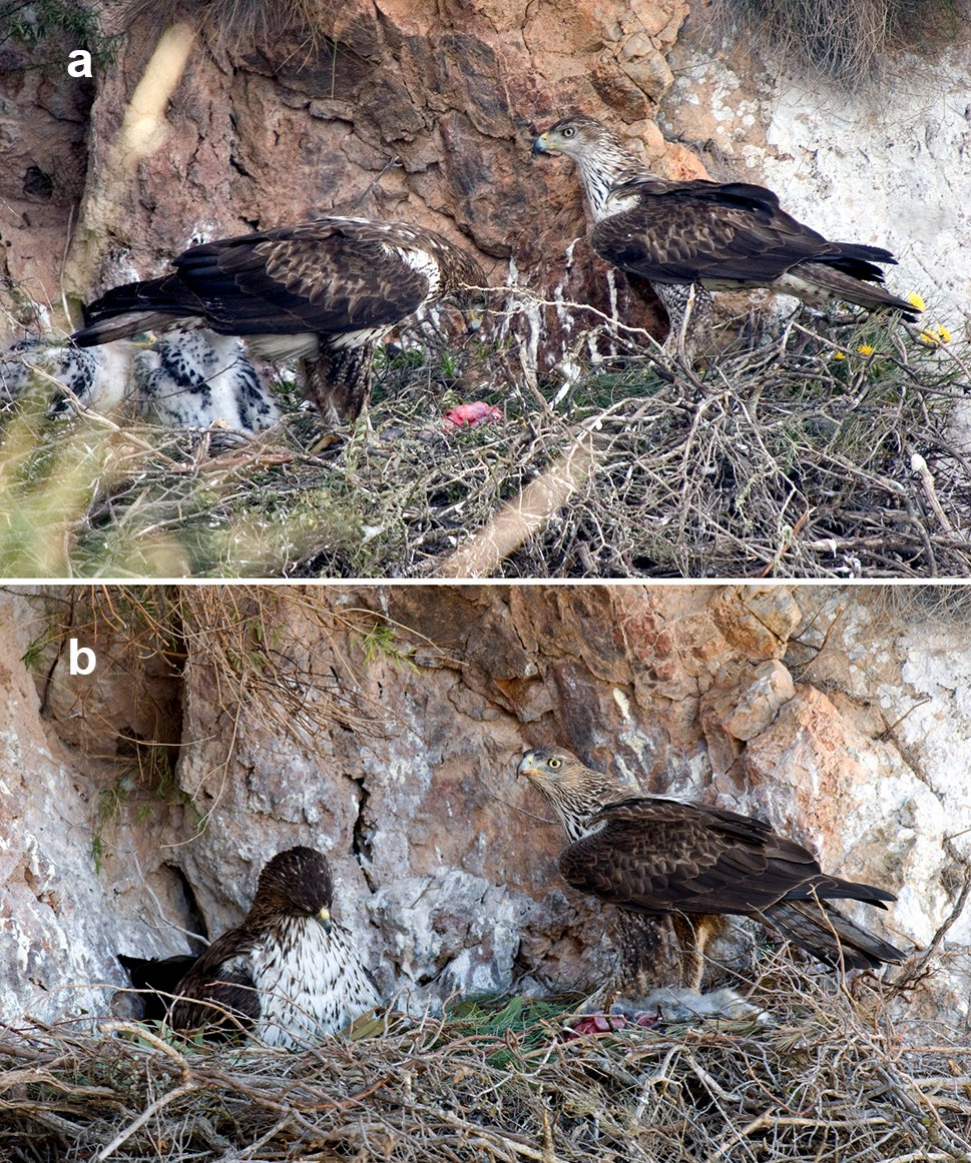
An adult male photographed during the 2008 breeding season (**a**) and a different male at the same nest during the 2012 breeding season (**b**). In both cases, the nest was occupied by the same female. Note the differences in the pigmentation of the cheek, throat and leg-feathers (see coloration patterns in^69^).

Capture-recapture methods involving identification based on photographs is recognized as a reliable method to monitor wildlife populations and assess ecological aspects such as population size and structure, survival, site-fidelity, occupancy, lifetime reproductive success and other variables^61–65^. Following previous studies on vultures^66^ and raptors, including Bonelli’s Eagle^61,67,68^, we took photographs of individual Bonelli’s Eagles using camera traps placed on rock perches^68^ and a digital camera mounted on a digiscoping adaptor attached to a spotting scope in a hide^66^. Territorial Bonelli’s Eagles could be recognized from these photographs of perched individuals (Fig. 1) using variation in plumage colour (cheek, general colour of the breast and neck), and especially in the pattern of pigmentation (throat, and particularly the leg-feathers; see^69^. The ability to recognize individuals on this basis persisted from year to year^68^. Using these photographs, we could use individual identifications to assess their behavior as a possible surrogate for the degree of their investment in nest-building. We identified all of the individuals from the 16 pairs. In total, 32 individuals (16 females and 16 males), belonged to the intensive monitoring pairs per year, were identified with certainty during the study period.

For analytical purposes, the monitoring season was divided into 16 weeks during the pre-laying period (weeks 1–16, counting backwards from the moment of egg-laying). The pre-laying period spanned the time between the last pre-dispersal flights of the young Bonelli’s Eagles of the previous year^70,71^, the beginning of courtship and nest-building in mid-October, up to the moment of egg-laying^59^. We determined the onset of laying by direct observation (i.e., onset of incubation and changeovers). Laying dates were recorded with a maximum error of ± 1 day.

Using our individual identifications, the experience of each known individual in a territory was determined by the number of consecutive years spent in the same territory, provided that the individual concerned was seen to be installed in the territory when first observed^63^. On the other hand, nest quality was quantified as the number of times that the nest has been used in the past. In this sense, we considered high quality nests when they were occupied more than five years while that low quality nests were those nests with occupation rates lower to four years. According to our field experience, the low quality nests of Bonelli's Eagles can be identified by their relatively small size compared to the high quality nests, which reach larger dimensions due to their reuse rate (authors unpubl. data). In addition, the monitored territories were visited at least four times post-laying to record the number of fledglings and to gather data on productivity. Nestlings observed at ≥ 50 days old were assumed to have fledged successfully^72,73^.

### Data analysis

We tested five hypotheses in total (Table 1). For hypotheses 1–2 and 4, we first applied a generalized linear mixed model (GLMM) to assess the factors determining the amount of material (number of branches) carried to the nest by parents and to explore whether the investment of mates in nest-building could act as an honest signal informing to the partners on their parental quality. To test these hypotheses, we quantified the daily rates of nest-material supply as the response variable under a negative binomial distribution. We included the bird’s sex, week, experience and nest quality as possible predictors of nest-building investment. For hypothesis 3a–3b, we performed a second GLMM using the daily rates of hard and fresh nest-material supply respectively as the response variable under a negative binomial distribution and the sex and the weeks as predictors. To account for possible correlation effects between the factors in the data, sex identity was included as a random factor.

**Table 1:** Hypotheses proposed in this study on the nest-building behaviour in Bonelli’s Eagles, expected results, and a summary of the main results obtained.

Hypothesis	Expected results	Observed results
1. A high effort of the males and females in the construction of the nests could indicate their qualities as good builders to their partners	There are differences in nest building effort between sexes	Males and females did not show differences in nest-building effort
2. The amount of material delivered to the nest increases as the date of the egg-laying approaches	The delivery of material by both sexes increased throughout the pre-laying period	The amount of material delivered to the nest by both sexes does not increase as the pre-laying period progresses: there are no differences between sexes, there is no relationship with the weeks
3a.The amount of hard material supplied to the nest varied throughout the pre-laying period	The amount of material supplied to the nest is higher in the first weeks of the pre-laying period due to its greater functionality. There are differences in the types of materials provided to the nests between sexes	The amount of hard material delivered to the nest varied throughout the pre-laying period: no differences were found between sexes, but there were differences with the weeks
3b. The amount of fresh material supplied to the nest varied throughout the pre-laying period	The amount of fresh material added to the nest is higher as the laying date approaches due to its functionality. There are differences in the contribution of fresh materials to the nests between sexes	The amount of fresh material delivered to the nest varied throughout the pre-laying period: no differences were found between sexes, but there were differences with the weeks
4. Nest-building investment depends on individual experience and nest quality	The more experienced individuals contributed higher amounts of material than the less experienced ones. High-quality nests receive more material than low-quality nests	Nest-building effort was determined by experience and the nest quality: the most experienced individuals contributed higher amount of material in low quality nests, while the less experienced individuals contributed similar amounts of material in high and low quality nests
5. Females reproductive investment depends on male’s nest-building effort	The nest building effort of the male acts as an honest signal that informs their mates of their parental quality: the females adjust their reproductive effort in response to the male’s nest-building effort	We did not find a relationship between the effort of supply of material by the males and an increase in the breeding quality index (BQI)

For hypothesis 5, we performed a third GLMM using the breeding quality index (hereafter, BQI) as a response factor under a normal distribution. BQI was defined as an individual’s ability to produce offspring compared with the average success of others in the same year. BQI was calculated as the difference between the number of eaglets fledged for a particular individual/territory and the average number of eaglets in the monitored territories in the same year^74^. In this analysis, male nest-building investment was considered as a predictive factor determining productivity. Sex identity was again considered as a random factor. All statistical analyses were performed with R 4.0.4^75^. The GLMM’s were analysed with glmer.nb function and lme4 package^76^. Statistical significance was set at *P* < 0.05.

### Ethic statements

Bird-photographing procedures, camera trapping and monitoring of Bonelli’s Eagles were conducted under permits and following the protocols approved by the competent Regional Government of Region of Murcia (Resolución AUF/2020/0107). All the work was conducted in accordance with relevant national and international guidelines, and conforms to all legal requirements in compliance with the Ethical Principles in Animal Research.

## Results

**Figure 2:**
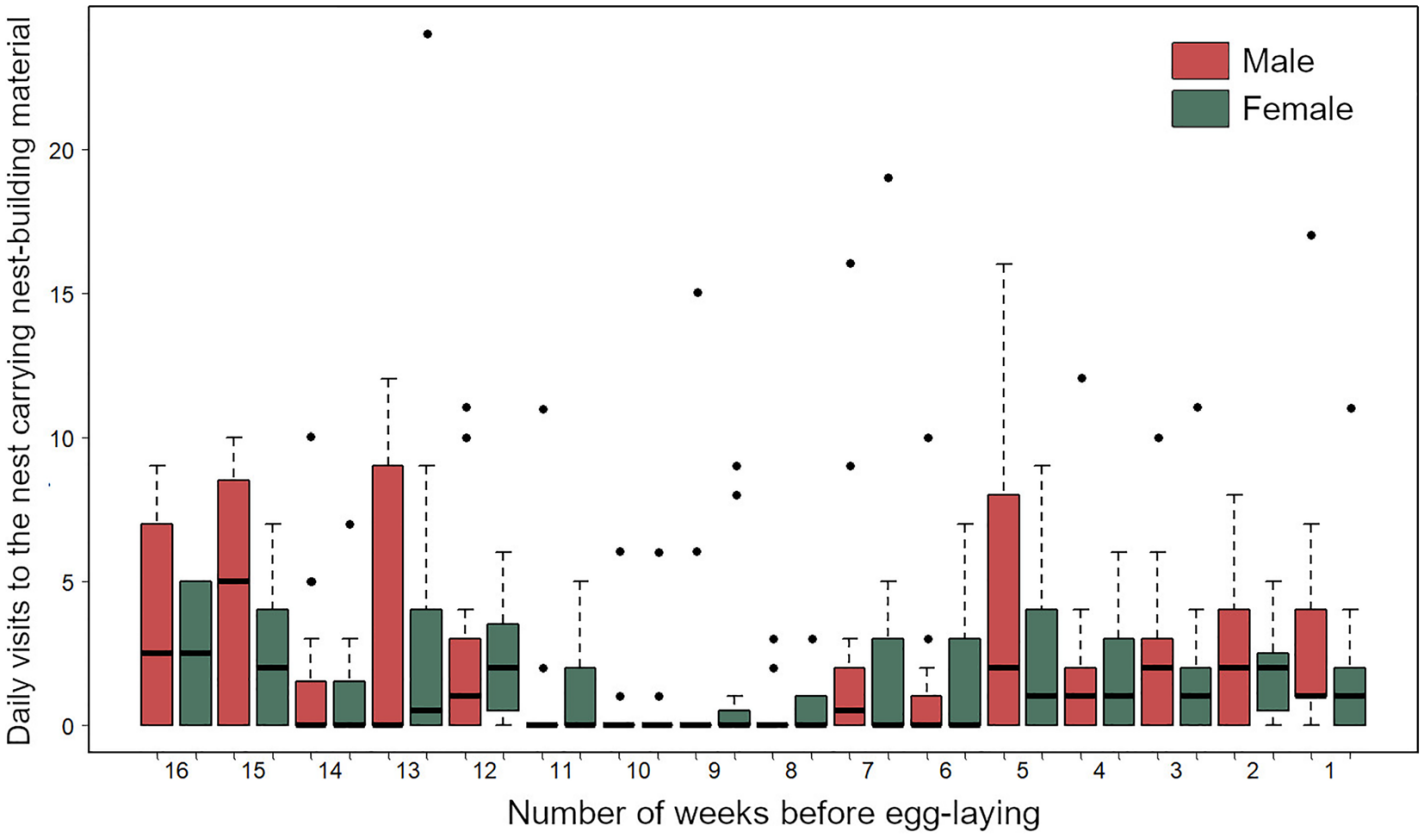
Tukey box plot for the provisioning of nest-building material during the pre-laying period by male and female Bonelli’s Eagles. The line within the box represents the median, the horizontal lines are the first and third quartiles (50% of the observations fall between the two, i.e., are in the box). Vertical lines depict intervals including other data up to 1.5 times the interquartile distance, and points represent outlying data.

Our observations showed that Bonelli’s Eagles selected their nest-site and began bringing hard and fresh material up to four months prior to egg-laying. The main materials used for nest construction were hard and fresh branches, principally of Aleppo Pine, with a small number of shrub species (Table 2). During the pre-laying period, males (mean: 2.01; 95% CI 1.42–2.83) were not more active than females (mean: 1.64; 95% CI 1.17–2.30) in their construction activity (Table 3). The supply of material to the nest began 16 weeks prior to egg-laying. In both sexes, there were two peaks in construction activity: the first, between 112 and 78 days prior to egg-laying (October and early November); and the second, between 35 days prior to egg-laying and clutch completion (end December and January). In contrast, the supply of nest-material was very low between 77 and 36 days prior to egg-laying (Fig. 2).

**Table 2:** The nest-building investment by Bonelli’s Eagles in 16 territories in southeastern Spain in terms of the type of material supplied to the nest per day.

Plant species	Fresh material (%)	Hard material (%)
*Pinus halepensis*	303 (63.65)	3 (0.99)
*Stipa tenacissima*	9 (1.90)	6 (2.00)
*Olea europaea*	5 (1.05)	0
*Anthyllis cytisoides*	1 (0.21)	0
*Ephedra fragilis*	2 (0.42)	0
*Pistacia lentiscus*	32 (6.72)	0
*Chamaerops humilis*	1 (0.21)	10 (3.31)
*Retama sphaerocarpa*	29 (6.10)	1 (0.33)
*Rosmarinus officinalis*	1 (0.21)	0
Unidentified	93 (19.53)	282 (93.37)
Total items	476 (61.18)	302 (38.82)
Males investment (mean, 95% CI)	1.30 (0.96–1.65)	0.89 (0.65–1.13)
Females investment (mean, 95% CI)	1.13 (0.78–1.47)	0.65 (0.41–0.89)

Mean, 95% CI, number, and percentages (in brackets) of sticks and branches of the different plant species (N = 778) of green (fresh) and non-green (hard) material, supplied by both sexes.

**Table 3:** Results of generalized linear mixed models for testing the five hypotheses examined.

Hypothesis	Response variable	Explanatory variable	Estimate	SE	*z* value	*P*
Hypothesis 1	Nest material	Sex (female)	− 0.200	0.217	− 0.925	0.355
Hypothesis 2	Nest material	Sex (female)	− 0.197	0.220	− 0.896	0.371
		Week	0.007	0.021	0.328	0.743
Hypothesis 3a	Hard nest material	Sex (female)	− 0.256	0.258	− 0.993	0.321
		Week	− 0.097	0.026	− 3.792	< 0.001
Hypothesis 3b	Fresh nest material	Sex (female)	− 0.154	0.282	− 0.546	0.585
		Week	0.084	0.022	3.742	< 0.001
Hypothesis 4	Nest material	Experience	0.120	0.038	3.108	< 0.001
		Nest quality (low)	1.265	0.551	2.295	< 0.05
		Experience * Nest quality (low)	− 0.040	0.060	− 0.671	0.502

			Estimate	SE	*t* value	*P*
Hypothesis 5	Breeding quality index (BQI)	Male nest-building investment	0.001	0.009	0.198	0.846

The values of the z and t statistics are also shown. Mean values are shown with their 95% confidence intervals, SE (Standard Error) and *P* values for each variable.

**Figure 3:**
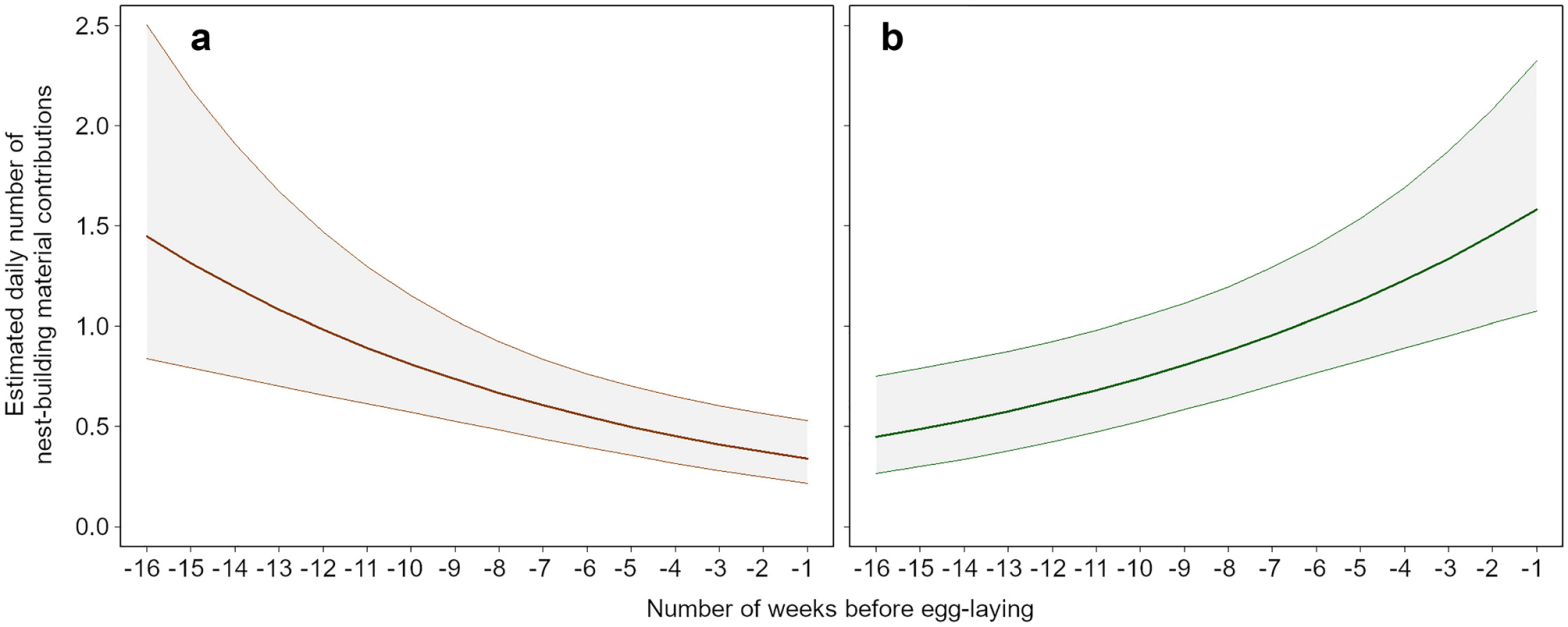
Estimated daily number of nest-building material contributions in relation to number of weeks before egg-laying (weeks − 16 to − 1). The plot on the left represents the estimated daily number of hard material supplied by both sexes to the nest throughout pre-laying period (**a**). The plot on the right represents the estimated daily number of fresh material supplied by both sexes to the nest throughout pre-laying period (**b**).

Overall, the amount of material delivered to the nest did not increase as the pre-laying period advanced (Table 3). In fact, we did not find differences between sexes, nor a relationship with the weeks. However, considering the type of material, Bonelli’s eagles showed a negative delivery rate of hard material and positive delivery rate of fresh material during the weeks (Table 3, Fig. 3). The peak of supply of hard material took place between weeks 16–12 prior to egg-laying and later decrease, while a greater amount of fresh material (green plants) was brought during the last five weeks prior to egg-laying (Fig. 3).

**Figure 4:**
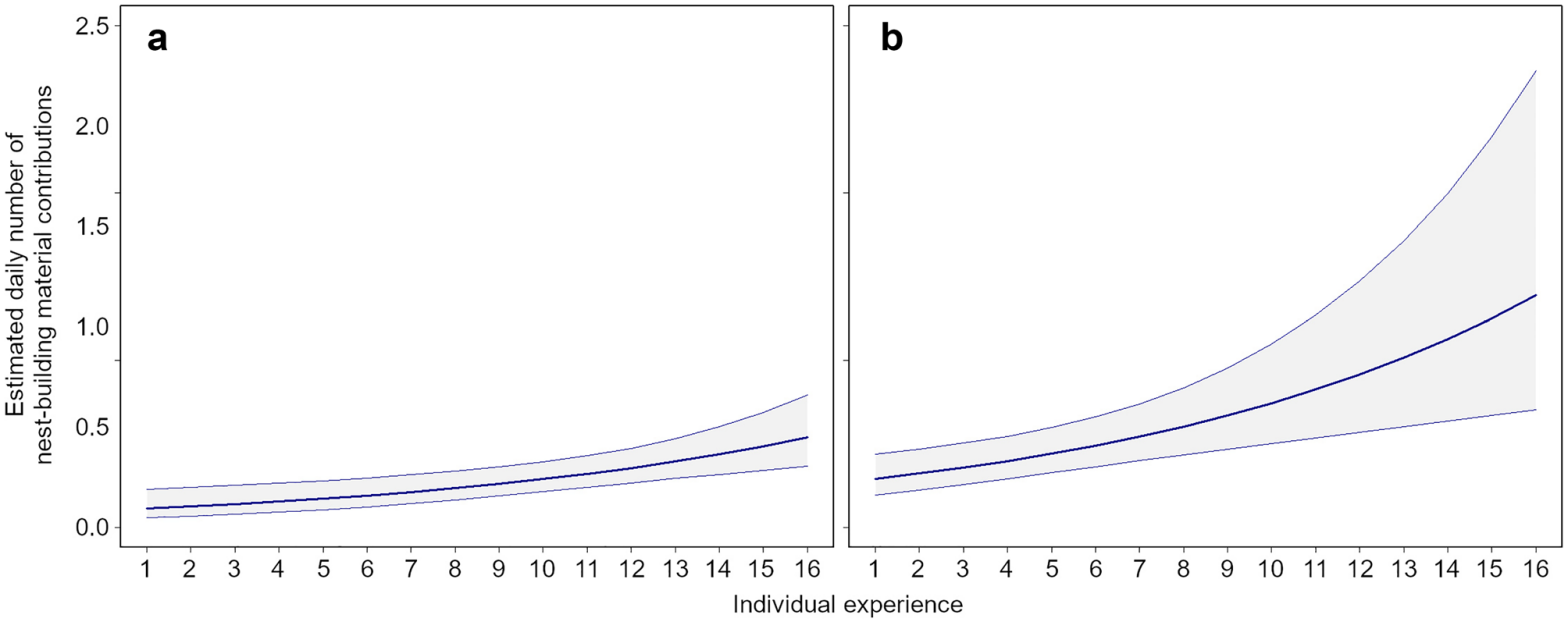
Estimated daily number of nest-building material contributions in relation to individual experience (expressed as number of consecutive years that an individual occupies the same territory when the first installation in the territory is observed; 1–16 years). The plot on the left represents the estimated daily number of material delivered by partners to the high-quality nests (**a**). The plot on the right represents the estimated daily number of material delivered by partners to the low-quality nests (**b**).

Our model (with two additive factors) shows that the nest-building effort of parents during pre-laying was significantly related either to their level of breeding experience at the territory and to the nest quality: the most experienced individuals contributed higher amount of material to the nests a lesser number of times used in the past (low quality nests), while the less experienced individuals contributed similar amounts of material in high and low quality nests (Table 3, Fig. 4). However, the interaction between the two factors (experience and nest quality) was not significant (*P* = 0.502). In addition, there were no significant relationships between the amount of material carried to the nest by males and the BQI (Table 3).

## Discussion

Behavioural studies exploring the function of nest-building behaviour in biparental species are scarce^43^. In most raptors, both partners invest in nest construction^60,77,78^. Thus, if investment in nest building is a consistent trait in either sex, it is likely that both partners use their mate's nest building effort to assess their parental quality^13,43^. In this way, the nest may indicate parental quality, experience or genetic quality, and therefore females could benefit from mating with good nest-building males^13^. Previous studies describing bi-parental care in Bonelli’s Eagles have shown a sex-biased specialization in parental duties^48,79^. Females invested significantly more effort than males in gathering nest-material during the incubation and offspring attendance period^48^. In contrast, our results showed that both sexes invested a balanced distribution of nest building effort during the courtship period. This result may be due to that both sexes use nest building contributions as a signal of their quality, and both sexes spur on the other sex to contribute more to nest building^13^. Our results, therefore, do not lend weight to Hypothesis 1.

In some raptors where both partners contribute in the supply of material to the nest, the pair may increase their effort as the laying date approaches^60,80^. In some species the parents carry material to the nest throughout the entire breeding season (even after the chicks fledge^9^). In Bearded Vultures *Gypaetus barbatus*, for example, males invest more effort in nest-building than females, showing a peak of construction activity between 4 and 2 weeks prior to egg-laying^60^. Given that this activity may represent an important effort in time and energy^1^, this behaviour would help the females to avoid an excessive drain on energy which would affect the optimal physical condition required for reproduction^81^. These studies showed a progressive increase in material delivered to the nest throughout the pre-laying period. We would expect similar behaviour in Bonelli’s Eagles, as we suggested in Hypothesis 2. However, our results did not show a temporal positive trend when the delivered rate of material was considered; instead of this, eagles showed a bimodal trend in the effort of material delivered to the nest during the courtship period. This result is related to the type of material delivered to the nest, which is related to the next hypotheses (3a and 3b).

Our results showed temporal changes in the types of material delivered to the nest, although no differences were found between the sexes. Bonellli’s Eagles delivered larger quantities of fine and coarse dry branches in the early visits to the nest and during the start of nest-building, whereas the supply of green material tended to increase as the laying date approached (Fig. 3). The fact that eagles delivered sticks and branches to nest several weeks before green plant material might be related to: (1) the need to create a larger nest structure, including size, thickness, mass and cup volume, which can influence the nest’s structural and thermal properties and so, buffer the impact of adverse environmental conditions on the development of embryos and nestlings^18,19^, and (2) signal nest occupancy by increasing the visibility of the nest-site to conspecifics and competitors (e.g., Golden Eagles, *Aquila chrysaetos*^17,44^). At the same time, previous studies have shown that green material brought to the nest can regulate the nest temperature and may help to decrease ectoparasite and pathogen loads^47,82,83^ and improve breeding success^12^. Although fresh nest-material was gathered from vegetation rich in resins, including nine plant species, the most abundant plant delivered to the nests was greenery from pines (Table 2). Pines are characterized by a high level of aromatic compounds, particularly ß-pinene, highly repellent for insects^12,24^. In Bonelli’s Eagle nests, the presence of ectoparasites (mainly blow fly larvae) has been described, which can directly affect the offspring mortality and reduce the breeding success of the host^55^. On the other hand, neither were any inter-sexual differences observed with regard to the type of nest-material gathered (hard and fresh). This finding is consistent with the results of^60^, who found that in Pyrenean Bearded Vultures, there were no observed inter-sexual differences concerning the amount supplied of either of the two common nest-materials used (branches and wool). Therefore, our results partially support Hypotheses 3a and 3b.

Breeding experience could be a decisive factor determining the investment effort of parents^44,84^. For example, this factor could influence individual decisions relating to nest material choice^33,36^ and the amount of material gathered for nest construction^34^. In this way, younger eagles, with less experience of nest construction, should supply lower amounts of sticks and branches than older, more experienced eagles^34^. At the same time, in some nest builders’ species, nest-building behaviour can be mainly influenced by nest characteristics^50,85^. However, the role of nest characteristics and nest-building effort has been scarcely explored in some nest reuser species, such as storks^9^ or raptors^44^. Our model shows that the nest-building effort was determined by the experience of the individual and by nest quality (Table 3). This result is striking, initially, it would be expected that experienced individuals would invest a greater amount of material in the nests most used in the past, that is, in those larger nests. For nest reuser species, the nest size could be an indicator of individual and/or nest-site quality^9^. In addition, in several species, nest size and nest building activity has been associated with the condition of nest-building males, and females in some cases^35^. However, our results show that the experienced individuals contributed higher amounts of material to the nests less used in the past (low quality nests). This result could be related to: (1) the need to invest in the maintenance of the least used nests, whose dimensions are smaller than those of the most used nests, in order to increase their decoration, size and conspicuousness from the air, even at large distances, avoiding possible territorial conflicts with close neighbors (‘‘signal-function’’ hypothesis^11^). According to this hypothesis, a group of nests located in the same territory should be conspicuous and widely dispersed within the same territory^12^.Thus, the need to increase the conspicuousness of the nests could act as a signaling medium and a reliable threat against Bonelli’s Eagles and others competitors that breed in the vicinity^17,44^. And (2) the age of the individual, and consequently its ability or experience as a builder, could increase the visibility of the nests and its function of signaling and reliable threat, being minimum for youngest individuals and peaked for individuals in prime age (10–12 years old), as suggested for the black kite *Milvus migrans*^44^. According to these authors, this behavioral pattern could reveal the viability, the territory quality and the conflict dominance of the signaler. Therefore, nests’ properties could have important consequences to encourage experience-related variation in Bonelli’s Eagle nest-building effort. Accordingly, our results support the Hypothesis 4.

Previous studies have shown that both sexes may signal their condition, health, or parental quality to mates by building large or elaborate nests or by intense nest-building activity^13,43^. At first glance, one might expect that Bonelli’s Eagles’ investment in nest-building could act as an honest signal informing mates about their parental quality; therefore females would adjust their reproductive investment based on the nest-building investment exhibited by their mates. Nevertheless, our analysis did not find a relationship between male nest-building investment and consequent reproductive performance. This result could be explained to: (1) Bonelli's Eagles often successfully produce 1–2 fledglings^56^, so it is hard to expect significant results of hypothesis related with BQI, as variability of such data is rather small; and (2) the task of nest construction does not fall mainly on males, so their effort would not constitute an honest signaling that inform the females of their quality as good builders, and therefore there would be no observed increase in BQI. In this respect, our results are not consistent with either the DAH^30^ or PCH^41^, thus rejecting our fifth hypothesis.

## Conclusions

Our findings show that there are no differences in male and female investment in nest building during the pre-laying period. Male nest-material provisioning rates therefore do not have a positive impact on reproductive success, therefore, does not act as an honest signal to indicate their parental quality to their mates. Nest-building behavior in the early and late stages of nest-building, and the fact that nest-building effort is determined by individual experience and nest quality, supports the idea of signaling nest occupancy to conspecifics and competitors and the decrease of ectoparasite loads during the pre-laying period.

## Supplementary Information

Supplementary Information.

## Data Availability

All data generated or analysed during this study are included in this published article (and its Supplementary Information files).
